# Liquid Organic Hydrogen Carrier Concepts and Catalysts for Hydrogenation and Dehydrogenation Reactions

**DOI:** 10.3390/molecules29204938

**Published:** 2024-10-18

**Authors:** Gerardo Cabrera, Malka Mora, Juan P. Gil-Burgos, Renso Visbal, Fiderman Machuca-Martínez, Edgar Mosquera-Vargas

**Affiliations:** 1 Centro de Excelencia en Nuevos Materiales (CENM), Universidad del Valle, Santiago de Cali 760032, Colombia; cabrege@gmail.com (G.C.); renso.visbal@correounivalle.edu.co (R.V.); fiderman.machuca@correounivalle.edu.co (F.M.-M.); 2 Grupo de Investigación en Síntesis Organometálica y Catálisis (GISIOMCA), Departamento de Química, Universidad del Valle, Santiago de Cali 760032, Colombia; malka.mora@correounivalle.edu.co (M.M.); juan.gil.burgos@correounivalle.edu.co (J.P.G.-B.); 3 Escuela de Ingeniería Química, Universidad del Valle, Santiago de Cali 760032, Colombia; 4 Grupo de Transiciones de Fase y Materiales Funcionales, Departamento de Física, Universidad del Valle, Santiago de Cali 760032, Colombia

**Keywords:** liquid organic hydrogen carrier (LOHC), catalysts, hydrogenation and dehydrogenation reactions

## Abstract

Background: The issue of renewable energy (RE) source intermittency, such as wind and solar, along with the geographically uneven distribution of the global RE potential, makes it imperative to establish an energy transport medium to balance the energy demand and supply areas. A promising energy vector to address this situation is hydrogen, which is considered a clean energy carrier for various mobile and portable applications. Unfortunately, at standard pressure and temperature, its energy content per volume is very low (0.01 kJ/L). This necessitates alternative storage technologies to achieve reasonable capacities and enable economically viable long-distance transportation. Among the hydrogen storage technologies using chemical methods, liquid organic hydrogen carrier (LOHC) systems are considered a promising solution. They can be easily managed under ambient conditions, the H_2_ storage/release processes are carbon-free, and the carrier liquid is reusable. However, the evolution of the proposals from the carrier liquid type and catalyst elemental composition point of view is scarcely studied, considering that both are critical in the performance of the system (operational parameters, kinetic of the reactions, gravimetric hydrogen content, and others) and impact in the final cost of the technology deployed. The latter is due to the use of the Pt group elements (PGEs) in the catalyst that, for example, have a high demand in the hydrogen production sector, particularly for polymer electrolyte membrane (PEM) water electrolysis. With that in mind, our objective was to examine the evolution and the focus of the research in recent years related to proposals of LOHCs and catalysts for hydrogenation and dehydrogenation reactions in LOHC systems which can be useful in defining routes/strategies for new participants interested in becoming involved in the development of this technology. Data sources: For this systematic review, we searched the SCOPUS database and forward and backward citations for studies published in the database between January 2011 and December 2022. Eligibility criteria: The criteria include articles which assessed or studied the effect of the type of catalyst, type of organic liquid, reactor design(s)/configuration(s), and modification of the reactor operational parameters, among others, over the performance of the LOHC system (de/hydrogenation reaction(s)). Data extraction and analysis: The relevant data from each reviewed study were collected and organized into a pre-designed table on an Excel spreadsheet, categorized by reference, year, carrier organic liquid, reaction (hydrogenation and/or dehydrogenation), investigated catalyst, and primary catalyst element. For processing the data obtained from the selected scientific publications, the data analysis software Orbit Intellixir was employed. Results: For the study, 233 studies were included. For the liquid carrier side, benzyltoluene and carbazole dominate the research strategies. Meanwhile, platinum (Pt) and palladium (Pd) are the most employed catalysts for dehydrogenation reactions, while ruthenium (Ru) is preferred for hydrogenation reactions. Conclusions: From the investigated liquid carrier, those based on benzyltoluene and carbazole together account for over 50% of the total scientific publications. Proposals based on indole, biphenyl, cyclohexane, and cyclohexyl could be considered to be emerging within the time considered in this review, and, therefore, should be monitored for their evolution. A great activity was detected in the development of catalysts oriented toward the dehydrogenation reaction, because this reaction requires high temperatures and presents slow H_2_ release kinetics, conditioning the success of the implementation of the technology. Finally, from the perspective of the catalyst composition (monometallic and/or bimetallic), it was identified that, for the dehydrogenation reaction, the most used elements are platinum (Pt) and palladium (Pd), while, for the hydrogenation reaction, ruthenium (Ru) widely leads its use in the different catalyst designs. Therefore, the near-term initiatives driving progress in this field are expected to focus on the development of new or improved catalysts for the dehydrogenation reaction of organic liquids based on benzyltoluene and carbazole.

## 1. Introduction

One of the greatest challenges of this century is reducing the environmental impact of anthropogenic activities, which requires significant changes in how we produce, distribute, store, and utilize energy. The majority of the consumed electrical energy is derived from fossil fuels; only 27.3% originates from renewable energy sources, with 8.7% being generated by variable renewable energy (VRE) sources, such as photovoltaic solar energy (PV) and wind energy [1]. With the rapid depletion of fossil fuels and the worsening environmental pollution caused by their consumption, there is a significant need to efficiently use energy and explore appropriate technologies that can replace existing ones, thereby enabling the sustainable development of our economy and society [2].

The challenge of intermittency in renewable energy sources (RESs), such as solar and wind, makes it imperative to store the generated energy to help stabilize these fluctuations. Furthermore, considering the land availability, the potential for harvesting RESs is limited in highly industrialized regions like Central Europe, Japan, South Korea, or Eastern China, while it is abundant in places like Iceland, Patagonia, or Western Australia [3]. Therefore, to utilize these RESs on a massive scale, conversion facilities should preferably be situated in regions with a high RES supply, where the generated energy must subsequently be stored and transported through energy carriers to these high-demand energy sites [4]. In this context, regions characterized by a constrained RES potential and a current net import of fossil energy carriers are likely to depend on low-carbon energy carrier imports in the future, shaping a new global energy market (export and import) [5]. In such a scenario, electrical energy not only needs to be stored on a large scale but also transported over long distances that clearly exceed the spans covered by a “classic” power line, considering economic limitations [4]. Hence, it is vital to establish an energy transport medium to balance the energy demand and supply areas [6].

A promising energy vector to address the aforementioned situation is hydrogen (H_2_), which is regarded as a clean energy carrier for various mobile and portable applications and can be utilized without greenhouse gas emissions [7,8,9]. The H_2_ molecule holds a high energy content per unit mass (120 MJ/kg), approximately three times that of oil. Unfortunately, at standard pressure and temperature, its energy content per unit volume is very low (0.01 kJ/L). Even at a pressure of 70 MPa, as currently used in fuel cell electric vehicles’ (FCEVs) commercial hydrogen storage tanks, H_2_ provides 1.3 kWh/L compared to gasoline’s 8.8 kWh/L [10]. This implies the need for alternative storage technologies to achieve reasonable capacities.

**Scheme 1:**
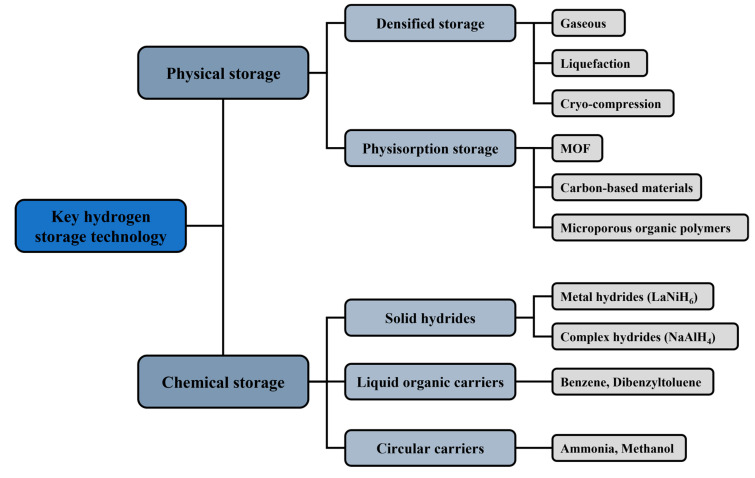
Classification of hydrogen storage technologies.

Hydrogen storage technologies can be broadly categorized into two main groups, physical methods and chemical methods (see Scheme 1), each with their own advantages and disadvantages. The first group includes dense hydrogen storage (compressed gas and liquid) and physisorption. Compressed hydrogen is the simplest form of storage, yet the hydrogen density per unit volume is low (42.2 kg-H_2_/m^3^ at 69 MPa), high pressures are required, and high-pressure leaks and embrittlement-related issues can occur. Liquid hydrogen has the highest hydrogen density per unit volume (70.8 kg-H_2_/m^3^), but extremely low temperatures (−253 °C) are needed to convert hydrogen into liquid form, demanding substantial energy consumption and challenging long-term storage or long-distance transport [11]. Meanwhile, in physisorption, hydrogen molecules are adsorbed onto the surface of an adsorbent (storage medium) due to intermolecular forces between the adsorbate (hydrogen) and the adsorbent. Materials in this category include carbon nanotubes, activated carbon, zeolites, and metal–organic frameworks (MOFs). These materials offer advantages in terms of their reversibility and relatively fast kinetics. However, the storage capacity is low (<5% by weight at room temperature), and the carrier materials are heavy, requiring low temperatures and high pressures for increased storage capacities [12,13].

On the other hand, in the second group, hydrogen molecules are dissociated into atoms and integrated into the chemical structure of the material. Metal hydrides have been extensively researched but still present issues regarding costs, weight, operating temperatures, charge–discharge kinetics, and the formation of unwanted gases during desorption [14]. Among cyclic carriers, methanol has been considered a promising candidate, where hydrogen can be released through thermolysis, steam reforming, and partial oxidation [15]. However, its use raises environmental concerns due to the CO_2_ release when utilized or directly decomposed. Furthermore, CO_2_ separation also consumes significant energy (an amine solution consumes around 1.1 kWh/kg-CO_2_) [16]. Finally, liquid organic hydrogen carrier (LOHC) storage systems are deemed a promising solution, as they can be easily handled under ambient conditions, storage/release processes are carbon-free, and the carrier liquid is non-depleting and reusable [14]. Moreover, it is envisioned as a significant option for hydrogen storage and long-distance or intercontinental transportation [17,18,19].

Therefore, the LOHC system consists of a pair of organic compounds, one rich in hydrogen (LOHC+) and one deficient in hydrogen (LOHC−). Hydrogen is stored through the conversion of LOHC− to LOHC+ in a catalytic hydrogenation reaction. Subsequently, hydrogen is released by converting LOHC+ back to LOHC− through a catalytic dehydrogenation [20,21,22]. Research on hydrogen-carrying liquid organic compounds focused mainly on identifying suitable compounds from liquid aromatic hydrocarbons such as benzene, toluene, and naphthalene [23,24]. Some molecules are even compatible with the existing fuel infrastructure and are not considered hazardous for transportation. Molecules like toluene are already key chemicals in the petrochemical industry. Hydrogen-rich LOHCs can also be stored under atmospheric and ambient pressure and temperature conditions, a clear advantage compared to, for example, liquefied ammonia [25]. However, the high dehydrogenation reaction temperature (around 300 °C) and the slow reaction rate of these aromatic compounds limit their large-scale application [23,24]. As for the catalyst, there is a preference for those based on platinum-group elements (PGEs) for the hydrogenation reaction [26], which are expensive and scarce. On the other hand, dehydrogenation catalysts require wet conditions but lead to the release of a considerable amount of H_2_ gas and are subject to deactivation by the liquid product [25].

However, researchers have focused their efforts on discovering potential carriers capable of reversible hydrogen storage under relatively mild temperature conditions, as well as on developing efficient catalytic systems (lowering activation energy and controlling kinetics) [27]. To date, excellent reviews discussing advancements in the search for new organic liquids for reversible hydrogen storage can be found [28,29], as well as the current state of progress in catalyst development for hydrogenation [26] and/or dehydrogenation processes [30]. However, to the best of our knowledge, the study of the evolution of the proposals from the carrier liquid type and catalyst elemental composition point of view have not been reported, considering that both are critical in the performance of the system (operational parameters, kinetic of the reactions, gravimetric hydrogen content, and others) and impact in the final cost of the technology deployed. The latter is due to the use of the Pt group elements (PGEs) in the catalyst that, for example, have a high demand in the hydrogen production sector, particularly for polymer electrolyte membrane (PEM) water electrolysis. With that in mind, the main aim of this work is to examine the evolution and the focus of the research in recent years related to proposals of LOHCs and catalysts for hydrogenation and dehydrogenation reactions in LOHC systems which can be useful in defining routes/strategies for new participants interested in becoming involved in the development of this technology.

## 2. Results and Discussion

### 2.1. Organic Hydrogen Carrier Liquids

**Figure 1:**
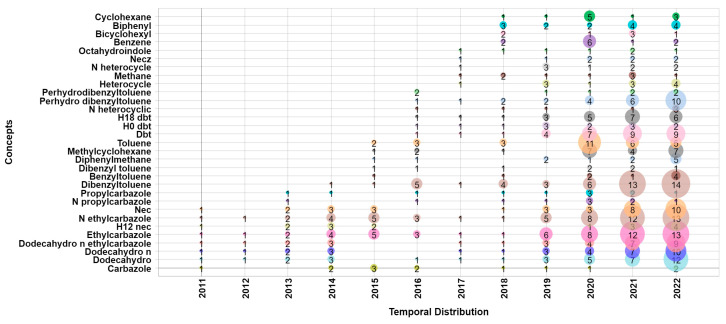
Concept/descriptor graph over time for the topic of organic liquids (extracted from Intellixir software (https://carlac.intellixir.fr/cenm, accessed on 16 October 2024) based on the 233 selected publications).

**Figure 2:**
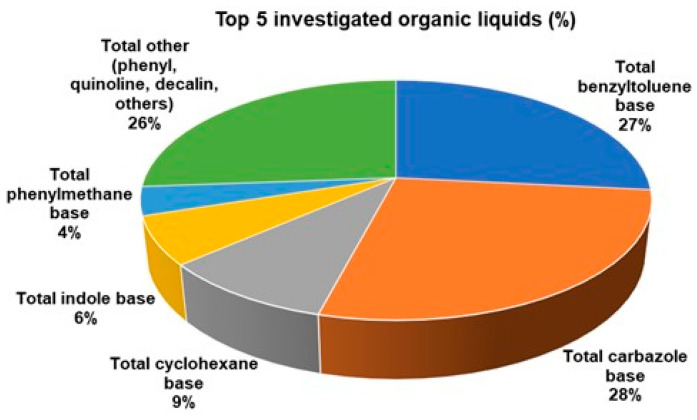
Percentage distribution for the top 5 investigated organic liquids. Source: Own elaboration based on data from the 233 selected publications.

Recently, the most studied LOHC compounds include benzene and cyclohexane [31,32], toluene and methylcyclohexane (MCH) [33,34,35], naphthalene and decalin [36,37], N-ethylcarbazole and perhydro-N-ethylcarbazole (H0-NEC and H12-NEC) [38,39], and dibenzyltoluene and perhydrodibenzyltoluene (H0-DBT and H18-DBT) [40,41] (see Table 1), respectively. However, when representing the temporal evolution of concepts related to organic liquids from the 233 selected publications (see Figure 1), a strong and growing trend can be observed in concepts associated with the base compound benzyltoluene (dibenzyltoluene, H0-DBT, perhydro-dibenzyltoluene, H18-DBT, and DBT, among others), and carbazole (N-ethylcarbazole, NEC, dodecahydro, and 12H-NEC, among others). Regarding this, a more detailed analysis of the data (Figure 2) shows that DBT and NEC together represent 55% of the total scientific research, surpassing the base organic liquids cyclohexane (9%), indole (6%), and phenylmethane (4%) by a wide margin. A recent techno-economic feasibility study showed that the H18-DBT/DBT pair system stores more H_2_ at a lower cost than the H12-NEC/NEC and toluene/MCH systems [42].

The development of alternative LOHC molecules remains critical for decreasing the heat demand and temperature levels during dehydrogenation, especially if the dehydrogenation enthalpy can be reduced while maintaining the benefits of low toxicity, adequate abundance, thermophysical properties (e.g., melting and flash points), and H_2_ storage capacity. DBT and BT are already commercially available as heat transfer fluids, although not at a scale comparable to the emerging hydrogen economy. Conversely, MCH and toluene are key components of the chemical industry, making them abundantly available, along with cyclohexane and benzene. However, benzene is a known carcinogen, and, like toluene, it exhibits severe toxicity and is highly flammable [25]. Accordingly, the following sub-sections provide a concise overview of three primary groups of compounds that have been the subject of extensive research within the scientific literature. These include aromatic *N*-heterocyclic compounds, homocyclic aromatic compounds, and oxygen-containing compounds.

#### 2.1.1. Aromatic *N*-Heterocyclic Compounds

**Figure 3:**
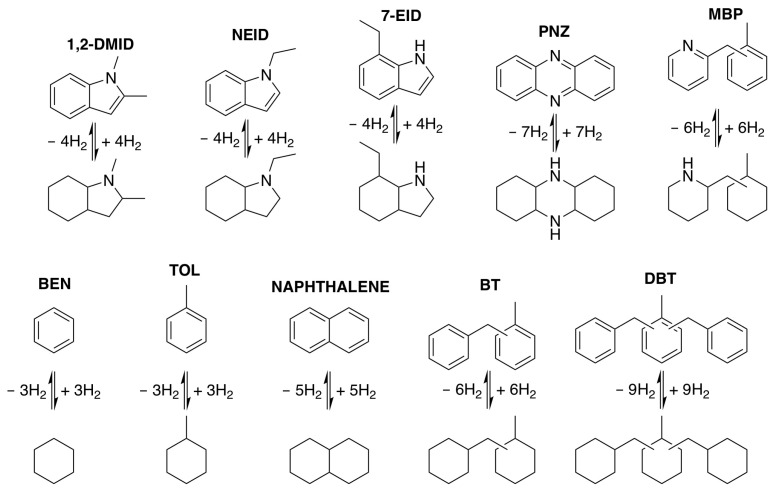
Common aromatic *N*–heterocyclic compounds used in LOHC systems.

Compounds containing nitrogen are among the most promising LOHCs due to their high gravimetric hydrogen densities and favorable hydrogen release kinetics. Some of the most relevant derivatives are described in Figure 3. The presence of N atoms in carbocyclic compounds (5.3–7.3% by weight) reduces the endothermicity of dehydrogenation compared to the corresponding homocyclic compounds due to the weaker C-H bond strength adjacent to the N atom, as N-H is weaker than the C-H bond adjacent to C and C-H. Therefore, hydrogen production can be achieved through dehydrogenation at a lower temperature. For this purpose, various nitrogen-containing systems, such as the dodecahydro-6,7-benzindole (hydrogenated form)/6,7-benzindole LOHC system, have been studied recently due to their lower reaction enthalpy compared to DBT (54 vs. 65 kJ mol^−1^ H_2_). Earlier in 2015, Li et al. [44] reported the catalytic activity of 5 wt% of Ru- and Pd/Al_2_O_3_ catalysts which promoted the full hydrogenation of 2-MID to 8H-2MID (Figure 3) under different temperatures. In this study, two species were proposed as key kinetically stable intermediates. Derivatives containing heteroatoms, such as nitrogen, offer significant advantages in terms of the thermodynamics and kinetics in hydrogenation/dehydrogenation processes in liquid organic hydrogen carrier (LOHC) systems. However, a major disadvantage of these systems is that the majority of derivatives possess high melting points. For instance, phenazine (PNZ), which exhibits a favorable hydrogen storage capacity (7.2 wt%), possesses a sufficiently elevated melting point (174–177 °C), thereby constraining its utility as an LOHC. Conversely, indole and its derivatives (1-MID, 2-MID, 1,2-DMID, NEID, and 7-EID), which display relatively low melting points (55–90 °C), exhibit a comparatively diminished hydrogen storage capacity between 5.2–5.8 wt% (see Table 1 and Figure 3) [24]. One of the lowest reaction enthalpies is exhibited by H12-NEC/NEC or H12-NPC/NPC LOHC (50.5 kJ/mol H_2_), driving the catalyst development for this molecule. Most of these molecules have been proposed before, and research focuses on developing suitable catalysts for efficient and selective dehydrogenation, although none of these compounds are available on a notable production scale [25,27].

Other examples include mono- and dimethylquinolines, as well as substituted quinolines and pyridines, which were also proposed due to their lower dehydrogenation enthalpy (56–62 kJ/mol H_2_). However, some substituted quinolines and pyridines exhibit a higher dehydrogenation enthalpy (greater than 65 kJ/mol).

#### 2.1.2. Homocyclic Aromatic Compounds

**Figure 4:**
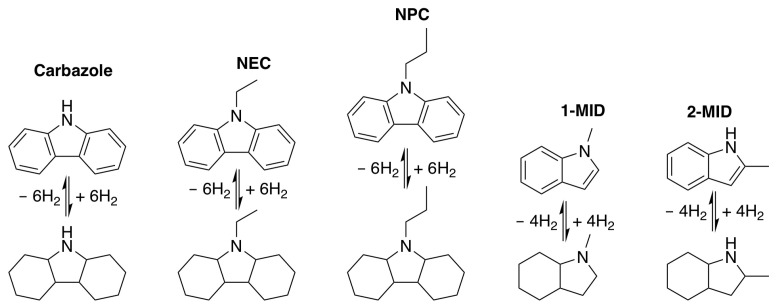
Common homocyclic aromatic compounds used in LOHC systems.

The initial LOHC studies focused on polycyclic hydrocarbons due to their advantages, such as the high gravimetric hydrogen content (5% by weight, 8% by weight), high volumetric hydrogen storage capacities (>60 g H_2_/L), and reversibility between hydrogenation and dehydrogenation. Simple aromatic compounds like benzene and toluene were extensively investigated in the 1980s as the first LOHCs (see Figure 4) [27]. More recently, compounds like dibenzyltoluene (DBT) and benzyltoluene (BT) have led the research in this group. However, BT is gradually replacing DBT due to its equivalent hydrogen storage capacity but with a reduced viscosity, higher reaction rates, and a lower formation of secondary products. BT has a higher vapor pressure compared to the H18-DBT/H0-DBT system, which is beneficial during dehydrogenation due to the reduction in the resulting hydrogen partial pressure. This reduction increases the driving force for dehydrogenation, allowing for lower dehydrogenation temperatures [25].

Among other proposals, it has been shown that light cycle oil (LCO) from fluid catalytic cracking could store up to 5.3% by weight of H_2_ and could be considered as a potential low-cost LOHC. Eutectic mixtures of biphenyl (biphenyl/diphenylmethane) have also been proposed to store nearly 7.0% by weight of H_2_. Another work of research has focused on decalin as a potential LOHC [25,27].

#### 2.1.3. Compounds Containing Oxygen

Compounds containing oxygen can benefit from the advantage of a lower dehydrogenation enthalpy, but the C–O bond breakage must be avoided. In this regard, an H_2_ capacity of 6.8% by weight has been reported using the eutectic mixture of biphenyl and diphenyl ether (another commercially known heat transfer fluid as Dowtherm A) with favorable dehydrogenation enthalpy. This is because the dehydrogenation enthalpies of ethers are in the same range as those of BT and other homocyclic aromatic compounds. Although significant amounts of cleavage products have been observed, these compounds have demonstrated a reversible cycle [25].

To increase the sustainability of LOHC systems, furfuryl alcohol with a dehydrogenation enthalpy of 77 kJ/mol H_2_ has been proposed, as well as trisphaeridine (Amaryllidaceae alkaloids) with an H_2_ capacity of 5.9% by weight and a capacity of 54 kJ/mol H_2_, and ethylene glycol/ethanol with a 5% by weight H_2_ storage capacity [25].

### 2.2. Catalysts for Hydrogenation and Dehydrogenation Reactions

**Figure 5:**
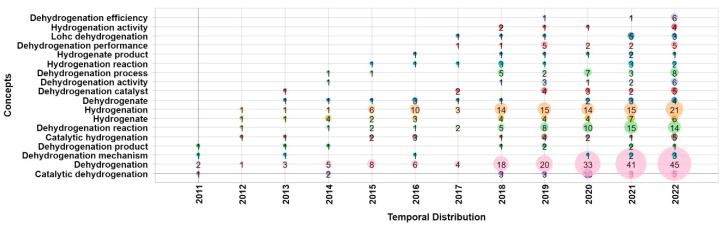
Temporal concept/descriptor graph for the topic of hydrogenation and dehydrogenation reactions, generated from Intellixir software based on 233 selected publications.

To accelerate the hydrogenation and dehydrogenation processes, catalysts are required to reduce the activation energy to a certain extent and control kinetics [45,46]. Although de/hydrogenation reactions are essential for the development of the LOHC technology, an analysis of the temporal evolution of concepts related to those reactions (see Figure 5) revealed a notable trend. Specifically, the dehydrogenation reaction exhibited a growing trend and a doubling in quantity and frequency relative to the hydrogenation reaction over the past three years. This is due to the complexity surrounding the dehydrogenation reaction in terms of working temperatures and slow kinetics. Consequently, there is an expectation that there will be greater interest in the development of catalysts to improve the dehydrogenation reaction.

Thus far, the most relevant catalysts are based on noble metals such as Ru, Pd, Pt, and Rh, which can work in both hydrogenation and dehydrogenation, and even in both reactions [26,30]; see Table 2 and Table 3. On the other hand, given the high cost and scarcity of these elements, another proposal that has emerged is the inclusion or doping with a second metallic component in single-metal catalysts [26,30]; see Table 4 and Table 5.

**Figure 6:**
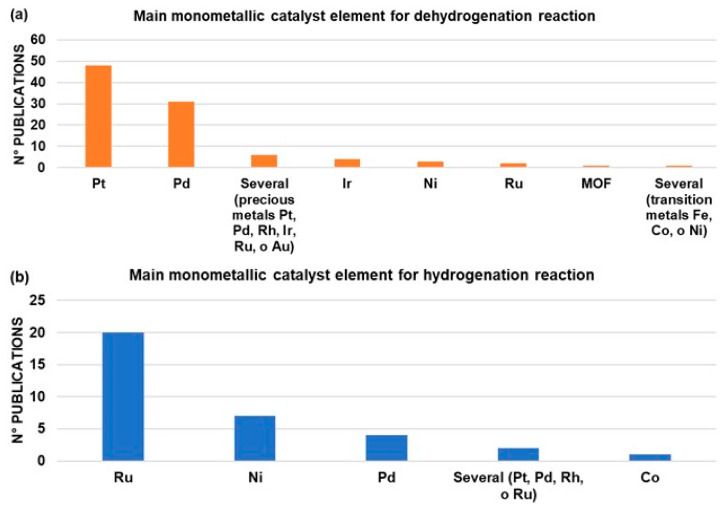
Distribution of elements in monometallic catalysts used in (**a**) dehydrogenation reactions and (**b**) hydrogenation reactions. “Several” indicates that the study evaluates various monometallic catalysts. Source: Own elaboration based on data from 233 selected publications.

**Figure 7:**
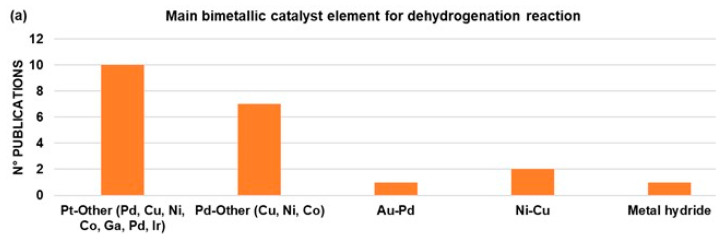

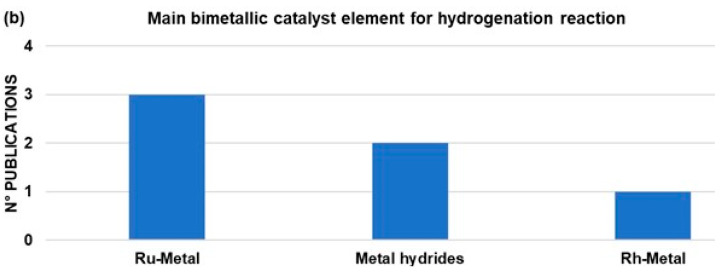
Distribution of elements in bimetallic catalysts used in (**a**) dehydrogenation reaction, and (**b**) hydrogenation reactions. Source: Own elaboration based on data from the 233 selected publications.

A more detailed description of the elements that comprise the types of catalysts (monometallic and bimetallic) in the de/hydrogenation reactions is provided in Figure 6 and Figure 7. In the context of dehydrogenation reactions, platinum (Pt) and palladium (Pd) are the most commonly utilized elements. Conversely, ruthenium (Ru) is a prominent element in the design of various catalysts for hydrogenation processes. It is crucial that we consider the prospective technical and economic implications of utilizing raw materials derived from the platinum-group elements (PGEs) for the deployment of LOHC systems within the context of the global hydrogen economy. This is due to the fact that these elements are costly and, in some instances, scarce. Furthermore, they are currently in high demand within the hydrogen production sector, particularly in the context of polymer electrolyte membrane (PEM) water electrolysis, which is regarded as a pivotal technology in the transition to a hydrogen-based economy [43,46,68,69].

Additionally, it has been discovered that selecting a suitable catalyst support to load active metallic components can further improve the catalytic performance of the catalyst and reduce costs by lowering the required loading amount [26,30]. In Ref [70], for example, for the hydrogenation of H0-DBT, a Pt catalyst was synthesized and supported on a range of materials, including Al_2_O_3_, hydroxylapatite (HAP), SBA-15, and activated carbon (C). The researchers proceeded to compare the catalytic activities of the aforementioned supported catalysts. The 5% Pt/Al_2_O_3_ catalyst was identified as the most effective, achieving the complete hydrogenation of H0-DBT within a short duration of 35 min. The catalytic activity of the various supports exhibited the following trend: alumina exhibited the highest level of activity, followed by SBA-15, HAP, and C, in descending order of effectiveness. In Ref. [71], the influence of diverse alumina morphologies as catalyst supports on the hydrogenation of H0-DBT was examined. The experimental findings indicated that the selection of alumina morphology had a notable impact on the hydrogenation of DBT, particularly with regard to the reaction kinetics. Furthermore, the enhanced dispersion of Pt on the catalyst surface increases its catalytic activity and facilitates efficient hydrogenation reactions. In contrast, in Ref. [72], a 3% Pt/Al_2_O_3_ catalyst was synthesized and the catalytic activities of Al_2_O_3_ treated with dielectric barrier discharge (DBD) plasma and untreated Al_2_O_3_ were compared. The researchers observed that the BDB plasma-treated Al_2_O_3_ exhibited enhanced catalytic activity in comparison to the untreated Al_2_O_3_. Additionally, the catalyst synthesized using O_2_ plasma demonstrated superior cyclic performance and stability over an extended period of time in comparison to other catalysts.

## 3. Methodology

This systematic review was conducted in accordance with the guidelines set forth in the Preferred Reporting Items for Systematic Reviews and Meta-Analyses (PRISMA) statement. A literature search was conducted on 10 March 2023, in the Scopus database (https://www.scopus.com, accessed on 10 March 2023) [73]. The search string utilized was as follows: TITLE-ABS-KEY ((LOHC OR {liquid organic hydrogen carrier})) AND PUBYEAR > 2010 AND PUBYEAR < 2023 AND (LIMIT-TO (DOCTYPE, “ar”)). The references were organized in Microsoft Excel. The initial screening was conducted based on the information presented in the titles and abstracts of the papers. The VOSviewer software (https://www.vosviewer.com, accessed on 5 April 2023) [74] was employed for the analysis and processing of scientific articles.

This systematic review encompassed all available studies that assessed or studied any component of the LOHC system. As evidenced by the search string, the inclusion criteria were articles published within the previous 12 years, written in English, and which assessed or studied the impact of various factors, including the type of catalyst, the type of organic liquid, reactor design and configuration, modification of operational parameters, and others, on the performance of the LOHC system (de/hydrogenation reaction(s)). The exclusion criteria included review articles, conference abstracts, book chapters, publications addressing cyclic liquid carriers (ammonia, methanol, formic acid, and formaldehyde) [19,27], and publications focused on the hydrogen value chain (production, storage, transport, and use), where the LOHC system is integrated as a comparative point with other hydrogen storage technologies (compression, liquefaction, etc.) for economic and prospective analyses (scenario simulation).

**Scheme 2:**
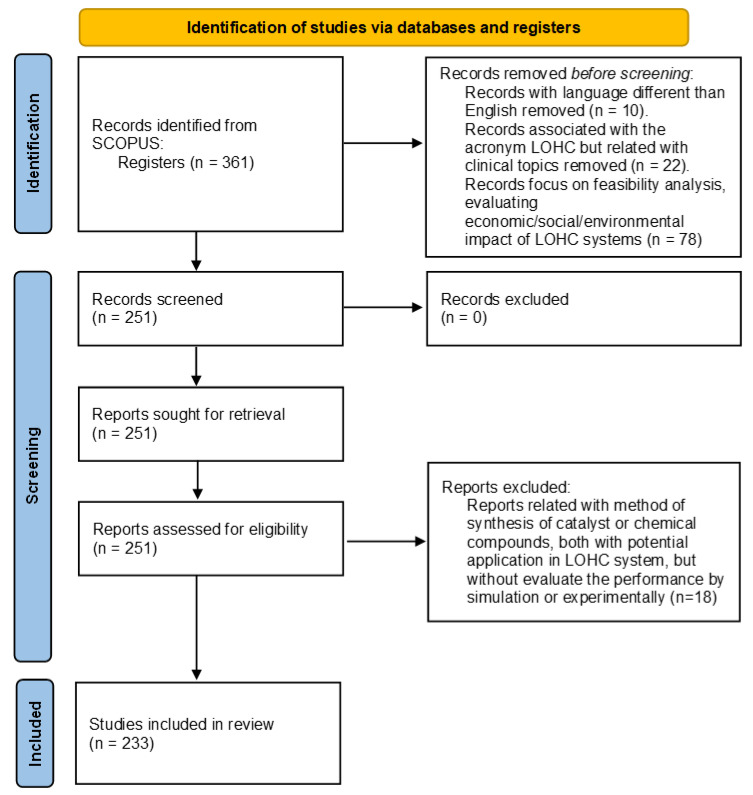
Flow diagram of the systematic review study selection process. Adapted from Ref. [76].

A total of 361 studies were initially identified through an electronic search; however, two studies written in Chinese and eight studies written in German were excluded due to their lack of relevance. Additionally, 22 studies associated with the acronym LOHC but related to clinical topics, and 78 studies focusing on feasibility analysis, evaluating the economic, social, and environmental impact of implementing an LOHC system as a hydrogen storage solution with a commercial liquid carrier or in conjunction with production hydrogen technology, were also excluded. Following the screening of titles and abstracts, 251 studies were deemed suitable for retrieval. Following a review of the full-text articles, 18 were excluded on the grounds that they are related to the synthesis of catalysts or chemical compounds, both of which have the potential to be applied in an LOHC system. However, they did not evaluate the performance of the system through simulation or experimentation, which is not relevant to the scope of this review. Following this process, 233 studies were deemed suitable for inclusion in the review. Subsequently, the pertinent data from each reviewed study were collated and organized into a pre-designed table on an Excel spreadsheet, categorized by reference, year, carrier organic liquid, reaction (hydrogenation and/or dehydrogenation), investigated catalyst, and primary catalyst element. The data obtained from the selected scientific publications were processed using the data analysis software Orbit Intellixir [75]. A flow diagram of the article selection process is shown in Scheme 2.

## 4. Conclusions

Hydrogen has emerged as one of the most extensively studied resources in the last two decades as an energy storage source, primarily due to its high energy density and the low environmental impact it generates. However, economically viable storage for transportation and use continues to be a challenge due to the low volumetric energy density and size of this element.

Among the technological options explored by the scientific community, liquid organic hydrogen carrier (LOHC) technology shows significant development potential. This is because LOHCs can be easily handled under ambient conditions, their storage/release processes are carbon-free, and the hydrogen carrier liquid is not consumed and can be reused. Furthermore, this technology is projected to be a significant option for hydrogen storage and transportation to intercontinental or long-distance markets.

Two key components for the implementation of LOHC technology, namely, the organic hydrogen carrier liquid and the catalysts for hydrogenation and/or dehydrogenation reactions, are subjects of extensive research and are considered hotspots in the field.

Among the available options for organic hydrogen carrier liquids, those based on benzyltoluene and carbazole are leading research efforts, accounting for more than 50% of the total scientific investigations, surpassing other organic liquids by a wide margin. However, proposals for organic liquids based on indole, biphenyl, cyclohexane, and cyclohexyl may emerge as contenders within the time frame considered in this review and should, therefore, be monitored for their development.

In the field of hydrogenation and dehydrogenation reactions, significant research efforts have been dedicated to enhancing the catalyst performance, particularly for dehydrogenation processes, which pose challenges due to the high temperature requirements and slow hydrogen release kinetics. The development of novel catalysts, especially those utilizing noble metals such as platinum (Pt), palladium (Pd), and ruthenium (Ru), has shown considerable promise. These metals, both in monometallic and bimetallic forms, have been explored extensively, particularly when supported on materials such as Al_2_O_3_ and SiO_2_. These supports provide structural stability and increase the surface area, thereby improving the catalytic activity. Given that these materials are part of the platinum-group elements (PGEs), which are expensive and currently in high demand in the hydrogen production chain, particularly in PEM water electrolysis technology, there is a need to assess the future technical and economic impact of PGE consumption in the implementation of LOHC systems within the global hydrogen economy.

Furthermore, their application in liquid organic hydrogen carrier (LOHC) systems highlights the potential for optimizing hydrogen storage and release mechanisms, making these systems more viable for industrial-scale hydrogen energy applications. Future research should continue focusing on tuning the catalytic properties of these metals, as well as exploring new support materials, to further improve the reaction efficiency, reduce the energy consumption, and accelerate the hydrogen release in dehydrogenation processes.

## Figures and Tables

**Table 1:** Physical parameters and hydrogen storage capacity of certain hydrogen-deficient LOHC carriers [43].

LOHCs	Melting Point (°C)	Boiling Point (°C)	H_2_ Storage Capacity wt%
Benzene (BEN)	5.5	80	7.2
Toluene (TOL)	−95	111	6.2
Naphthalene	80	218	7.3
Carbazole	245	355	6.7
N-ethylcarbazole (NEC)	69	378	5.8
N-propylcarbazole (NPC)	48	336	5.43
Benzyltoluene (BT)	−55	280	6.2
Dibenzyltoluene (DBT)	−39~−34	390	6.2
1-methylindole (1-MID)	−20	239	5.76
2-methylindole (2-MID)	57	273	5.76
1,2-dimethylindole (1,2-DMID)	55	260	5.23
N-ethylindole (NEID)	−17.8	253.5	5.23
7-ethylindole (7-EID)	14	230	5.23
2-(N-methylbenzyl)-pyridine (MBP)	−50.1~−40.2	291~293	6.15
Phenazine (PNZ)	174–177	357.2	7.2

**Table 2:** Catalytic hydrogenation performance of some monometallic catalysts for LOHC.

LOHCs	Catalysts	T (°C)	P_H2_ (MPa)	Time (h)	Conv ^a^ (%)	Yield ^b^ (%)	TOF (h^−1^)	Ref.
BEN	Ru/SBA-15	20	1	-	100	100	85.3	[43]
BEN	Ru/MOF	60	6	1.5	100	100	3200	[43]
BEN	4.2 wt% Ru/C-silica	110	8	0.53	100	99.8	37.7	[43]
BEN	Ru(0)-Zeolite-Y	22	0.28	1	100	100	1040	[43]
BEN	Ru/CNTs	80	4	0.5	100	99.97	6983	[43]
BEN	Pd/SiO_2_ (co-SEA)	150	7	6	84.1	84.1	-	[43]
TOL	Ni nanoflowers	140	5	0.5	100	100	-	[43]
TOL	Pd/SiO_2_ (co-SEA)	150	7	6	85.4	85.4	-	[43]
TOL	Pt (MP)/CeO_2_-A-400	100	0.5	3	90.8	90.8	-	[43]
NAP	Pt/WO_3_-500	70	3	1	100	100	-	[43]
NAP	Pd/HY-9.5	200	4	1	100	73.15	-	[43]
TEN	1 wt% Ni/Al_2_O_3_–YH_3_	150	10	5	-	95	-	[43]
MBT	0.5 wt% Ru/MgO	150	5	1.87	-	100	7680	[47]
MBT	5 wt% Ru/ZrO_2_	150	5	-	-	100	-	[48]
MBT	0.5 wt% Ru/Al_2_O_3_	175	10	0.104	-	53	-	[49]
DBT	0.5 wt% Ru/MgO	150	5	3.33	-	100	2880	[47]
DBT	Ni70/AlSiO-1/1	150	7	1.5	100	100	-	[43]
DBT	0.3 wt% Pt/Al_2_O_3_	270	3	1.42	-	100	-	[43]
DBT	5 wt% Pd/Al_2_O_3_	260	3	6	-	100	-	[43]
DBT	5 wt% Ru/Al_2_O_3_	170	5	0.772	-	100	-	[50]
DBT	Raney-Ni	170	7	30	-	100	-	[51]
DBT	Raney-Ni	170	0.8	18	-	-	-	[52]
DBT	Ni-based (NISAT 310)	170	2	3	-	100	-	[53]
DBT	Raney-Ni	170	0.8	10	-	100	-	[54]
DBT	Ru/Al_2_O_3_	130	3	16	-	100	-	[55]
NEC	Ru/pg-BC	130	6	1.17	100	99.41	-	[43]
NEC	Raney-Ni	180	5	1.3	-	86.2		[43]
NEC	Ni70/AlSiO-1/1	150	7	1.5	100	100	--	[43]
NEC	1.3 wt% Ru/YH_3_	130	7	2.5	100	100	-	[43]
NEC	5 wt% Ru/TiO_2_	130	7	-	-	95	-	[43]
NEC	Ru black	130	7	-	-	85	-	[43]
NEC	1.5 wt% Ru-Ni_1_Al_2_-LDO	150	8	1	100	100	-	[43]
NEC	1 wt%Ni/Al_2_O_3_–YH_3_	180	10	1.5	100	100	-	[43]
NEC	5 wt% Ru/LDH-3.9CNT	120	6	0.4	100	98.31	-	[43]
NEC	Ru/P25	150	7	24	100	92.4	-	[43]
NEC	Ru/anatase	150	7	24	100	95.7	-	[43]
NEC	Ru/Ni-Fe LDH	110	6	1.33	-	98.88	-	[43]
NPC	5 wt% Ru/Al_2_O_3_	150	7	0.5	-	100	-	[43]
NPC	Ni70/AlSiO-1/1	150	7	1	100	100		[43]

^a^: Conversion of LOHCs (hydrogen-deficient carriers); ^b^: yield of complete hydrogenation products.

**Table 3:** Catalytic dehydrogenation performance of some monometallic catalysts for LOHC.

LOHCs	Catalysts	T (°C)	P (MPa)	Time (h)	Conv ^a^ (%)	Yield ^b^ (%)	H_2_ Release (wt%)	Ref.
12H-MBT	3 wt% Pt/Al_2_O_3_ (decorated with S)	250	-	4	-	86.8	-	[56]
12H-MBT	5 wt% Pt/Al_2_O_3_ (doped with WO_x_)	250	-	3.5	-	75.9	-	[57]
18H-DBT	0.3 wt% Pt/Al_2_O_3_	310	0.1	2	-	95	-	[58]
18H-DBT	1 wt% Pd/C	290	-	-	-	97.6	-	[54]
18H-DBT	0.5 wt% Pt/Al_2_O_3_	300	-	1.67	-	60	-	[59]
18H-DBT	Pt/Al_2_O_3_	315	0.1	-	-	100	-	[60]
18H-DBT	0.3 wt% Pt/Al_2_O_3_	320	0.122	-	-	90	-	[61]
18H-DBT	1 wt% Pt/Al_2_O_3_	290	0.1	14	-	91.0	-	[62]
18H-DBT	5 wt% Pt/Al_2_O_3_	290	-	7	-	90.2	-	[52]
18H-DBT	0.3 wt% Pt/Al_2_O_3_	240	0.1	4	-	58.2	-	[63]
18H-DBT	5 wt% Pt/Al_2_O_3_ (doped with WO_x_)	270	-	3.5	-	64.8	-	[57]
18H-DBT	3 wt% Pt/Al_2_O_3_ (decorated with S)	250	-	4	-	86.8	-	[56]
12H-NEC	5 wt% Pd/NGC	180	0.1	10	100	98.72	5.76	[43]
12H-NEC	2.5 wt% Pt/SiO_2_-TiO(OH)_2_	180	0.1	7	100	97.9	5.75	[43]
12H-NEC	2.5 wt% Pd/LDHs-us	180	0.1	6	100	-	5.72	[43]
12H-NEC	1 wt% Pd-EU/KIT-6	190	0.1	6	100	100	-	[43]
12H-NEC	5 wt% Pd/Al_2_O_3_	180	0.1	4	100	100	-	[43]
12H-NEC	5 wt% Pt/Al_2_O_3_	180	0.1	5	100	100	-	[43]
12H-NEC	4 wt% Pd/SiO_2_	170		1.6	100	100	5.8	[43]
12H-NEC	2.5 wt% Pd/rGO-_EG_	170	0.1	12	100	84.61	5.49	[43]
12H-NEC	5 wt% Pt/TiO_2_	180	0.1	6	100	79	5.38	[43]
12H-NEC	2.32 wt% Pd/rGO	180	0.1	-	100	97.65	5.74	[43]
12H-NPC	1 wt% Pd/Al_2_O_3_-120	180	7	6	100	100	5.43	[43]
12H-NPC	3 wt% Pd@MIL-101	190	0.1	4	100	100	5.43	[43]

^a^: Conversion of LOHCs (complete hydrogenation carriers); ^b^: yield of complete dehydrogenation products.

**Table 4:** Hydrogenation performance of some bimetallic catalysts of LOHCs.

LOHCs	Catalysts	T (°C)	P (MPa)	Time (h)	Conv ^a^ (%)	Yield ^b^ (%)	Ref.
BEN	Pd-Ni/SiO_2_ (co-SEA)	150	7	6	99.9	99.9	[43]
BEN	Pd-Pt/SiO_2_ (co-SEA)	150	7	6	90.8	90.8	[43]
BEN	0.024 wt% Ru–1.00 wt% Ni/C	60	700 psi	2	100	100	[43]
BEN	Ru_0.56_Ni_0.44_/C	60	5.3	0.5	-	99.8	[43]
BEN	1 wt% Ru_2_Pt_1_ MIL-101	60	1	6	100	100	[43]
TOL	Pd-Ni/SiO_2_ (co-SEA)	150	7	6	99.9	99.9	[43]
TOL	Pd-Pt/SiO_2_ (co-SEA)	150	7	6	91.4	91.4	[43]
TOL	6 wt% Pt_1_Pd_1_/HBEA	150	7–12	2	100	100	[43]
TOL	Pt–Rh/MWNTs	20	1	3	100	100	[43]
DBT	Mg_2_NiH_4_	280	6	20	-	-	[64]
NAP	Ru/Ni/Ni(OH)_2_/C	100	4.48	1	-	>99	[43]
NAP	Ru/Ni/NiO/C	100	4.5	1	100	100	[43]
NAP	Ru/Co/Co_3_O_4_/C	100	4.5	0.8	100	100	[43]
NEC	5.0 wt% Ni_0.5_Ru_4.5_/pg-BC	130	6	1.17	100	99.06	[43]
NEC	5.0 wt% Co@Ru/NGC	130	6	1	100	99.1	[43]
NEC	Ru-Ni/P25	150	7	24	100	93	[43]
NEC	Ru-Ni/anatase	150	7	24	100	94.8	[43]
NEC	Ru_0.7_Ni_0.3_/SBA15	100	5	1.33	100	99.82	[43]
NPC	Ru_2.5_Ni_2.5_/Al_2_O_3_	150	4	0.5	100	100	[43]

^a^: Conversion of LOHCs (hydrogen-deficient carriers); ^b^: yield of complete hydrogenation products.

**Table 5:** Catalytic dehydrogenation performance of some bimetallic catalysts for LOHC.

LOHCs	Catalysts	T (°C)	P (MPa)	Time (h)	Conv ^a^ (%)	Yield ^b^ (%)	H_2_ Release (wt%)	H_2_ Evolution Rate mmol/g_met_/min	Ref.
CYH	10 wt% Ni_0.8_Cu_0.2_/ACC	350	-	10	25.78	-	-	39.45	[43]
CYH	10 wt% Ag-1 wt% Pd/ACC	300	-	7	-	-	-	7.5	[43]
CYH	10 wt% Ag-1 wt% Rh/ACC	300	-	6	-	-	-	12.34	[43]
CYH	10 wt% Ag-1 wt% Pt/ACC	300	-	6	-	-	-	13.36	[43]
CYH	5 wt% 1:4 Ag-Rh/Y_2_O_3_	300	0.1	4	-	-	-	400	[43]
CYH	5 wt%1:4 Ag-Rh/ACC	300	0.1	5	-	-	-	178.7	[43]
MCH	2.5wt%Pt_0.8_Ir_0.2_/Mg-Al-O	350	-	1.6	91.1	99.9	-	263.9	[43]
MCH	2.0 wt% Pt-0.5 wt% Sn/MgAleO-350	300	-	12	90.5	-	-	262.1	[43]
MCH	Pt-Cu/S-1	400	0.1	6	92.26	-	-	445.3	[43]
12H-MBT	Pt-Pd/Al_2_O_3_	250	0.1	6	-	99	-	-	[65]
18H-DBT	1 wt% Pt-1 wt% Pd/Al_2_O_3_	320	-	1.33	-	10	-	-	[66]
18H-DBT	Membranas basadas en Pd-Ag	350	0.4	-	-	-	-	-	[67]
12H-NEC	5 wt% PdCo/NGC	180	0.1	6	100	97.87	5.71	-	[43]
12H-NEC	Pd_3_ (3.75 wt%)-Ni_1_/SiO_2_	180	0.1	8	100	91.1	5.63	-	[43]
12H-NEC	Pd_3_ (3.75wt%)-Cu_1_/SiO_2_	180	0.1	8	100	83.11	5.47	-	[43]
12H-NEC	Pd_3_ (3.75wt%)-Au_1_/SiO_2_	180	0.1	8	100	94.9	5.7	-	[43]
12H-NEC	0.65 mol%Pd_1.3_–0.52 mol% Au1/rGO	180	0.1	4	100	100	5.79	-	[43]
12H-NEC	0.58 mol%Pd_1.3_–0.42 mol% Ru_1_/rGO	180	0.1	4	100	84.11	5.48	-	[43]
12H-NEC	Pd_1_ (2.5 wt%)-Co_1_/Al_2_O_3_	180	0.1	8	100	85.4	5.52	-	[43]
12H-NEC	Pd_4_Ni_1_/KIT-6	180	0.1	6	-	-	5.74	-	[43]
12H-NEC	Pd_1.2_Cu/rGO	180	0.1	7	100	100	5.79	-	[43]
12H-NPC	5 wt%Pd_1_-Ni_1_/Al_2_O_3_	180	6	7	100	100	5.43	-	[43]

^a^: Conversion of LOHCs (complete hydrogenation carriers); ^b^: yield of complete dehydrogenation products.

## Data Availability

No additional information is available for this study.

## References

[1] (2020). Renewables 2020 Global Status Report. https://www.ren21.net/wp-content/uploads/2019/05/gsr_2020_full_report_en.pdf.

[2] Liu C., Li F., Ma L.-P., Cheng H.-M. (2010). Advanced Materials for Energy Storage. Adv. Mater..

[3] Jorschick H., Bulgarin A., Alletsee L., Preuster P., Bösmann A., Wasserscheid P. (2019). Charging a Liquid Organic Hydrogen Carrier with Wet Hydrogen from Electrolysis. ACS Sustain. Chem. Eng..

[4] Niermann M., Drünert S., Kaltschmitt M., Bonhoff K. (2019). Liquid organic hydrogen carriers (LOHCs)—Techno-economic analysis of LOHCs in a defined process chain. Energy Environ. Sci..

[5] Hank C., Sternberg A., Köppel N., Holst M., Smolinka T., Schaadt A., Hebling C., Henning H.M. (2020). Energy efficiency and economic assessment of imported energy carriers based on renewable electricity. Sustain. Energy Fuels.

[6] Kojima H., Nagasawa K., Todoroki N., Ito Y., Matsui T., Nakajima R. (2023). Influence of renewable energy power fluctuations on water electrolysis for green hydrogen production. Int. J. Hydrogen Energy..

[7] Chang P.-L., Hsu C.-W., Lin C.-Y. (2012). Assessment of hydrogen fuel cell applications using fuzzy multiple-criteria decision making method. Appl. Energy.

[8] Hosseini S.E., Wahid M.A. (2016). Hydrogen production from renewable and sustainable energy resources: Promising green energy carrier for clean development. Renew. Sustain. Energy Rev..

[9] Biniwale R.B., Rayalu S., Devotta S., Ichikawa M. (2008). Chemical hydrides: A solution to high capacity hydrogen storage and supply. Int. J. Hydrogen Energy.

[10] Pasquini L. (2020). Design of Nanomaterials for Hydrogen Storage. Energies.

[11] Aziz M., Wijayanta A.T., Nandiyanto A.B. (2020). Ammonia as Effective Hydrogen Storage: A Review on Production, Storage and Utilization. Energies.

[12] Chamoun R., Demirci U.B., Miele P. (2015). Cyclic Dehydrogenation–(Re)Hydrogenation with Hydrogen-Storage Materials: An Overview. Energy Technol..

[13] Niaz S., Manzoor T., Pandith A.H. (2015). Hydrogen storage: Materials, methods and perspectives. Renew. Sustain. Energy Rev..

[14] Moradi R., Groth K.M. (2019). Hydrogen storage and delivery: Review of the state of the art technologies and risk and reliability analysis. Int. J. Hydrogen Energy.

[15] Goeppert A., Czaun M., Jones J.-P., Prakash G.S., Olah G.A. (2014). Recycling of carbon dioxide to methanol and derived products—Closing the loop. Chem. Soc. Rev..

[16] Bui M., Adjiman C.S., Bardow A., Anthony E.J., Boston A., Brown S., Fennell P.S., Fuss S., Galindo A., Hackett L.A. (2018). Carbon capture and storage (CCS): The way forward. Energy Environ. Sci..

[17] Niermann M., Timmerberg S., Drünert S., Kaltschmitt M. (2021). Liquid Organic Hydrogen Carriers and alternatives for international transport of renewable hydrogen. Renew. Sustain. Energy Rev..

[18] Zhang C., Song P., Zhang Y., Xiao L., Hou J., Wang X. (2022). Technical and cost analysis of imported hydrogen based on MCH-TOL hydrogen storage technology. Int. J. Hydrogen Energy.

[19] Abdin Z., Tang C., Liu Y., Catchpole K. (2021). Large-scale stationary hydrogen storage via liquid organic hydrogen carriers. iScience.

[20] Teichmann D., Arlt W., Wasserscheid P. (2012). Liquid Organic Hydrogen Carriers as an efficient vector for the transport and storage of renewable energy. Int. J. Hydrogen Energy.

[21] Teichmann D., Arlt W., Wasserscheid P., Freymann R. (2011). A future energy supply based on Liquid Organic Hydrogen Carriers (LOHC). Energy Environ. Sci..

[22] Sultan O., Shaw H. (1975). Study of Automotive Storage of Hydrogen Using Recyclable Liquid Chemical Carriers. [Catalytic Dehydrogenation of Naphthenes]. https://www.osti.gov/biblio/5000657.

[23] Choi I.Y., Shin B.S., Kwak S.K., Kang K.S., Yoon C.W., Kang J.W. (2016). Thermodynamic efficiencies of hydrogen storage processes using carbazole-based compounds. Int. J. Hydrogen Energy.

[24] Stepanenko S.A., Shivtsov D.M., Koskin A.P., Koskin I.P., Kukushkin R.G., Yeletsky P.M., Yakovlev V.A. (2022). N-Heterocyclic Molecules as Potential Liquid Organic Hydrogen Carriers: Reaction Routes and Dehydrogenation Efficacy. Catalysts.

[25] Perreault P., Van Hoecke L., Pourfallah H., Kummamuru N.B., Boruntea C.R., Preuster P. (2023). Critical challenges towards the commercial rollouts of a LOHC-based H2 economy. Curr. Opin. Green. Sustain. Chem..

[26] Kim T.W., Jeong H., Baik J.H., Suh Y.-W. (2022). State-of-the-art Catalysts for Hydrogen Storage in Liquid Organic Hydrogen Carriers. Chem. Lett..

[27] Cho J.-Y., Kim H., Oh J.-E., Park B.Y. (2021). Recent Advances in Homogeneous/Heterogeneous Catalytic Hydrogenation and Dehydrogenation for Potential Liquid Organic Hydrogen Carrier (LOHC) Systems. Catalysts.

[28] Niermann M., Beckendorff A., Kaltschmitt M., Bonhoff K. (2019). Liquid Organic Hydrogen Carrier (LOHC)—Assessment based on chemical and economic properties. Int. J. Hydrogen Energy.

[29] Aakko-Saksa P.T., Cook C., Kiviaho J., Repo T. (2018). Liquid organic hydrogen carriers for transportation and storing of renewable energy—Review and discussion. J. Power Sources.

[30] Jo Y., Oh J., Kim D., Park J.H., Baik J.H., Suh Y.W. (2022). Recent progress in dehydrogenation catalysts for heterocyclic and homocyclic liquid organic hydrogen carriers. Korean J. Chem. Eng..

[31] Koutsonikolas D., Kaldis S., Zaspalis V.T., Sakellaropoulos G.P. (2012). Potential application of a microporous silica membrane reactor for cyclohexane dehydrogenation. Int. J. Hydrogen Energy.

[32] Kou Z., Zhi Z., Xu G., An Y., He C. (2013). Investigation of the performance and deactivation behavior of Raney-Ni catalyst in continuous dehydrogenation of cyclohexane under multiphase reaction conditions. Appl. Catal. A Gen..

[33] Ali J.K., Rippin D.W.T. (1995). Comparing Mono- and Bimetallic Noble-Metal Catalysts in a Catalytic Membrane Reactor for Methylcyclohexane Dehydrogenation. Ind. Eng. Chem. Res..

[34] Ferreira-Aparicio P., Rodriguez-Ramos I., Guerrero-Ruiz A. (2002). On the Performance of Porous Vycor Membranes for Conversion Enhancement in the Dehydrogenation of Methylcyclohexane to Toluene. J. Catal..

[35] Oda K., Akamatsu K., Sugawara T., Sikuchi R., Segawa A., Nakao S.I. (2010). Dehydrogenation of Methylcyclohexane to Produce High-Purity Hydrogen Using Membrane Reactors with Amorphous Silica Membranes. Ind. Eng. Chem. Res..

[36] Hodoshima S., Arai H., Saito Y. (2003). Liquid-film-type catalytic decalin dehydrogeno-aromatization for long-term storage and long-distance transportation of hydrogen. Int. J. Hydrogen Energy.

[37] Jiang N., Rao K.S.R., Jin M.-J., Park S.-E. (2012). Effect of hydrogen spillover in decalin dehydrogenation over supported Pt catalysts. Appl. Catal. A Gen..

[38] Morawa Eblagon K., Tam K., Yu K.M.K., Zhao S.L., Gong X.Q., He H., Ye L., Wang L.C., Ramirez-Cuesta A.J., Tsang S.C. (2010). Study of Catalytic Sites on Ruthenium for Hydrogenation of N-ethylcarbazole: Implications of Hydrogen Storage via Reversible Catalytic Hydrogenation. J. Phys. Chem. C.

[39] Sotoodeh F., Smith K.J. (2010). Kinetics of Hydrogen Uptake and Release from Heteroaromatic Compounds for Hydrogen Storage. Ind. Eng. Chem. Res..

[40] Modisha P.M., Jordaan J.H.L., Bösmann A., Wasserscheid P., Bessarabov D. (2018). Analysis of reaction mixtures of perhydro-dibenzyltoluene using two-dimensional gas chromatography and single quadrupole gas chromatography. Int. J. Hydrogen Energy.

[41] Geburtig D., Preuster P., Bösmann A., Müller K., Wasserscheid P. (2016). Chemical utilization of hydrogen from fluctuating energy sources—Catalytic transfer hydrogenation from charged Liquid Organic Hydrogen Carrier systems. Int. J. Hydrogen Energy..

[42] Byun M., Lee A., Cheon S., Kim H., Lim H. (2022). Preliminary feasibility study for hydrogen storage using several promising liquid organic hydrogen carriers: Technical, economic, and environmental perspectives. Energy Convers. Manag..

[43] Zhang J., Yang F., Wang B., Li D., Wei M., Fang T., Zhang Z. (2023). Heterogeneous Catalysts in N-Heterocycles and Aromatics as Liquid Organic Hydrogen Carriers (LOHCs): History, Present Status and Future. Materials.

[44] Li L., Yang M., Dong Y., Mei P., Cheng H. (2016). Hydrogen storage and release from a new promising liquid Organic Hydrogen storage carrier (LOHC): 2-methylindole. Int. J. Hydrogen Energy.

[45] Dobereiner G.E., Crabtree R.H. (2010). Dehydrogenation as a Substrate-Activating Strategy in Homogeneous Transition-Metal Catalysis. Chem. Rev..

[46] Crabtree R.H. (2017). Nitrogen-Containing Liquid Organic Hydrogen Carriers: Progress and Prospects. ACS Sustain. Chem. Eng..

[47] Ouimet R.J., Glenn J.R., De Porcellinis D., Motz A.R., Carmo M., Ayers K.E. (2022). The Role of Electrocatalysts in the Development of Gigawatt-Scale PEM Electrolyzers. ACS Catal..

[48] Kim T.W., Kim M., Kim S.K., Oh H., Suh Y.-W. (2021). Remarkably fast low-temperature hydrogen storage into aromatic benzyltoluenes over MgO-supported Ru nanoparticles with homolytic and heterolytic H_2_ adsorption. Appl. Catal. B Environ..

[49] Kim T.W., Park S., Oh J., Shin C.-H., Suh Y.-W. (2018). Hydrogenation of the LOHC Compound Monobenzyl Toluene over ZrO_2_-supported Ru Nanoparticles: A Consequence of Zirconium Hydroxide’s Surface Hydroxyl Group and Surface Area. ChemCatChem.

[50] Leinweber A., Müller K. (2018). Hydrogenation of the Liquid Organic Hydrogen Carrier Compound Monobenzyl Toluene: Reaction Pathway and Kinetic Effects. Energy Technol..

[51] Kim T.W., Jeong H., Kim D., Park J.H., Suh Y.-W. (2022). Feasible coupling of CH_4_/H_2_ mixtures to H_2_ storage in liquid organic hydrogen carrier systems. J. Power Sources.

[52] Ali A., Rohini A.K., Noh Y.S., Moon D.J., Lee H.J. (2022). Hydrogenation of dibenzyltoluene and the catalytic performance of Pt/Al_2_O_3_ with various Pt loadings for hydrogen production from perhydro-dibenzyltoluene. Int. J. Energy Res..

[53] Ali A., Kumar G.U., Lee H.J. (2021). Investigation of hydrogenation of Dibenzyltoluene as liquid organic hydrogen carrier. Mater. Today Proc..

[54] Modisha P., Bessarabov D. (2020). Stress tolerance assessment of dibenzyltoluene-based liquid organic hydrogen carriers. Sustain. Energy Fuels.

[55] Ali A., Kumar G.U., Lee H.J. (2020). Parametric study of the hydrogenation of dibenzyltoluene and its dehydrogenation performance as a liquid organic hydrogen carrier. J. Mech. Sci. Technol..

[56] Heller A., Rausch M.H., Schulz P.S., Wasserscheid P., Fröba A.P. (2016). Binary Diffusion Coefficients of the Liquid Organic Hydrogen Carrier System Dibenzyltoluene/Perhydrodibenzyltoluene. J. Chem. Eng. Data..

[57] Jo Y., Wan Kim T., Oh J., Kim D., Suh Y.-W. (2022). Mesoporous sulfur-decorated Pt–Al_2_O_3_ for dehydrogenation of perhydro benzyltoluenes: Activity-favorable adsorption of reaction species onto electron-deficient Pt atoms. J. Catal..

[58] Kim C.H., Lee M.-W., Jang J.S., Lee S.H., Lee K.-Y. (2022). Enhanced activity of a WOx-incorporated Pt/Al_2_O_3_ catalyst for the dehydrogenation of homocyclic LOHCs: Effects of impregnation sequence on Pt–WOx interactions. Fuel.

[59] Auer F., Blaumeiser D., Bauer T., Libuda J., Wasserscheid P. (2019). Boosting the activity of hydrogen release from liquid organic hydrogen carrier systems by sulfur-additives to Pt on alumina catalysts. Catal. Sci. Technol..

[60] Modisha P., Garidzirai R., Güneş H., Erkey C., Bessarabov D. (2022). A Promising Catalyst for the Dehydrogenation of Perhydro-Dibenzyltoluene: Pt/Al_2_O_3_ Prepared by Supercritical CO_2_ Deposition. Catalysts.

[61] Naseem M., Usman M., Lee S. (2021). A parametric study of dehydrogenation of various Liquid Organic Hydrogen Carrier (LOHC) materials and its application to methanation process. Int. J. Hydrogen Energy.

[62] Rao N., Lele A.K., Patwardhan A.W. (2022). Optimization of Liquid Organic Hydrogen Carrier (LOHC) dehydrogenation system. Int. J. Hydrogen Energy.

[63] Shi L., Qi S., Smith K.J., Alamoudi M., Zhou Y. (2023). Dehydrogenation of the liquid organic hydrogen carrier perhydrodibenzyltoluene—Reaction pathway over Pt/ Al_2_O_3_. React. Chem. Eng..

[64] Mrusek S., Preuster P., Müller K., Bösmann A., Wasserscheid P. (2021). Pressurized hydrogen from charged liquid organic hydrogen carrier systems by electrochemical hydrogen compression. Int. J. Hydrogen Energy.

[65] Feng X., Jiang L., Li Z., Wu Y., Yuan B. (2022). Boosting the hydrogenation activity of dibenzyltoluene catalyzed by Mg-based metal hydrides. Int. J. Hydrogen Energy.

[66] Jorschick H., Geißelbrecht M., Eßl M., Bösmann A., Wasserscheid P. (2020). Benzyltoluene/dibenzyltoluene-based mixtures as suitable liquid organic hydrogen carrier systems for low temperature applications. Int. J. Hydrogen Energy.

[67] Modisha P., Gqogqa P., Garidzirai R., Ouma C.N.M., Bessarabov D. (2019). Evaluation of catalyst activity for release of hydrogen from liquid organic hydrogen carriers. Int. J. Hydrogen Energy.

[68] Wunsch A., Mohr M., Pfeifer P. (2018). Intensified LOHC-dehydrogenation using multi-stage microstructures and Pd-based membranes. Membranes.

[69] Cabrera Papamija G., Diosa Astaiza J.E., Mosquera Vargas E.E. (2024). Nanomateriales Carbonosos en Electrodos de Celdas PEM Utilizadas en la Electrolisis del Agua Para la Producción de Hidrógeno.

[70] Shi L., Qi S., Qu J., Che T., Yi C., Yang B. (2019). Integration of hydrogenation and dehydrogenation based on dibenzyltoluene as liquid organic hydrogen energy carrier. Int. J. Hydrogen Energy.

[71] Shi L., Zhou Y., Tan X., Qi S., Smith K.J., Yi C., Yang B. (2022). The effects of alumina morphology and Pt electron property on reversible hydrogenation and dehydrogenation of dibenzyltoluene as a liquid organic hydrogen carrier. Int. J. Hydrogen Energy.

[72] Shi L., Zhou Y., Qi S., Smith K.J., Tan X., Yan J., Yi C. (2020). Pt catalysts supported on H_2_ and O_2_ plasma-treated Al_2_O_3_ for hydrogenation and dehydrogenation of the liquid organic hydrogen carrier pair dibenzyltoluene and perhydrodibenzyltoluene. ACS Catal..

[73] Elsevier-Scopus. https://www.scopus.com.

[74] VOSviewer. https://www.vosviewer.com.

[75] Orbit Intellixir. https://carlac.intellixir.fr/cenm.

[76] Page M.J., McKenzie J.E., Bossuyt P.M., Boutron I., Hoffmann T.C., Mulrow C.D., Shamseer L., Tetzlaff J.M., Akl E.A., Brennan S.E. (2021). The PRISMA 2020 statement: An updated guideline for reporting systematic reviews. BMJ.

